# Is Dietary Iodine Intake Excessive According to the Theoretical Model of Healthy Dietary Intake Pattern in Pregnant Women and Schoolchildren: Water, Salt, or Food?

**DOI:** 10.3389/fnut.2021.770798

**Published:** 2021-12-10

**Authors:** Aline Carare Candido, Sarah Aparecida Vieira Ribeiro, Mariana de Souza Macedo, Edimar Aparecida Filomeno Fontes, Eliana Carla Gomes De Souza, Maria Sonia Lopes Duarte, Silvia Eloiza Priore, Maria do Carmo Gouveia Peluzio, Regina Célia Rodrigues de Miranda Milagres, Sylvia do Carmo Castro Franceschini

**Affiliations:** ^1^Department of Nutrition and Health, Universidade Federal de Viçosa, Viçosa, Brazil; ^2^Programa de Pós-Graduação em Ciência da Nutrição, Universidade Federal dos Vales do Jequitinhonha e Mucuri, Diamantina, Brazil

**Keywords:** iodine intake, pregnant women, school children, nutritional status, inadequacy

## Abstract

**Introduction:** Iodine deficiency during pregnancy can cause hypothyroidism and goiter; in schoolchildren, it can cause reduced intelligence quotient. In excess, iodine can cause thyroiditis, goiter, and Hashimoto's hypothyroidism. Currently, schoolchildren and pregnant women are classified as risk groups for excessive iodine intake and iodine deficiency, respectively. Thus, determining iodine from all sources of consumption is important for intervention planning.

**Objective:** To construct a theoretical model for the iodine intake of schoolchildren and pregnant women of a city in the Zona da Mata Mineira region, considering a healthy diet, salt consumption and water intake.

**Methodology:** The dietary iodine intake of pregnant women was analyzed based on a dietary iodine table compiled from an international database. A dietary plan was prepared following the Brazilian Food Guide. Iodine concentration of different salt brands sold in local establishments was checked, and drinking water samples from healthcare facilities were analyzed. A descriptive and exploratory statistical analysis was performed and the results were presented in absolute and relative frequencies, and measures of central tendency and dispersion.

**Results:** According to the proposed diet, pregnant women and schoolchildren would have a daily intake of 71.6 μg and 71 μg, respectively. Thirteen salt brands were evaluated, 69.2% complied with the legislation and the mean iodine content was 29.88 mg. The mean concentration of iodine in water was 25 μg iodine/liter and 14 μg iodine/liter, respectively, in summer and autumn. Considering the intake of food, salt, and drinking water according to the proposed dietary plan, the daily intake for pregnant women would be 279.5 and 253.5 μg for schoolchildren.

**Conclusion:** The daily iodine intake of schoolchildren and pregnant women according to this theoretical model was excessive, considering a healthy dietary pattern. This theoretical model can guide actions and public policies aimed at targeting all forms of iodine intake.

## Introduction

Iodine is an essential micronutrient for the synthesis of the thyroid hormones, Triiodothyronine (T3) and Thyroxine (T4). The amount of iodine required for the production of these hormones varies according to age and physiological state (1). Thus, the World Health Organization (WHO) recommends a daily iodine intake of 120 μg for schoolchildren and 150 μg for adolescents (over 12 years) and adults (2).

The recommended daily intake during pregnancy is higher (250 μg) due to body changes, fetal growth and development, and specific physiological changes such as thyroid hormone stimulation, hormone delivery to fetus, and increased glomerular filtration (urinary excretion of 30 to 50% of iodine) (2). This period is also marked by nutritional vulnerability given the energy needs for delivery and lactation, and fetal development (3).

Among pregnant women, a urinary iodine concentration lower than 150 μg is considered iodine deficiency. In schoolchildren, this concentration is <100 μg. Iodine deficiency in pregnant women causes hypothyroidism, goiter, and impaired fetal growth and poor brain development (2, 4). While in schoolchildren, it can culminate in significant reduction of intelligence quotient (IQ) and increased dropout rates (5). On the other hand, ingesting more than 300 μg of iodine is excessive for both groups, which can lead to thyroiditis, goiter, and Hashimoto's hypothyroidism (6).

In order to promote adequate iodine intake among the Brazilian population, salt iodization is mandatory by law in Brazil (Decree N° 39.814) since 1956. However, the implemented iodization range (20–60 ppm) was reduced in 2013 (15–45 ppm, Resolution N° 23) due to the high consumption of salt (12 mg/daily) among Brazilians (data from the Brazilian Institute of Geography and Statistics) (7, 8).

According to the National Survey on the Impact of Salt Iodization, 20.7% of schoolchildren presented urinary iodine levels between 200 and 299 μg/L (more than adequate) and 44.6% presented urinary iodine levels ≥300 μg/L (excessive). When stratified by sex, 49.7 and 40.8% of boys and girls, respectively, presented excessive iodine intake (9).

Studies have shown insufficient iodine intake in pregnant women, hence they are considered a risk group for iodine deficiency (3, 10, 11). From the above considerations, the study focused on two distinct groups, schoolchildren and pregnant women, the former with excessive iodine status and the latter with iodine deficiency. To plan effective interventions for these groups, iodine intake from all sources must be estimated. Therefore, the objective of this paper is to construct a theoretical model of iodine intake of schoolchildren and pregnant women in a city located in the Zona da Mata Mineira region, considering a healthy diet, salt consumption and water intake.

## Methods

### EMDI-Brazil

This work is part of the Multicenter Study of Iodine Deficiency (EMDI-Brazil) aimed at assessing the nutritional profile of iodine, sodium and potassium among mother and infant groups in Brazilian macro-regions.

### Assessment of Dietary Intake of Iodine

Dietary iodine intake was assessed based on a Dietary Iodine Table compiled from international databases (12).

The Estimated Energy Requirement (EER) for a schoolchild was calculated considering an 8-year-old boy. The adopted height and weight values were those of the 50th percentile of the World Health Organization (13). Accordingly, the EER of a schoolchild was 1.438 calories/day. For a pregnant woman, we used the energy recommendation for women aged 19–50 years, plus an additional value for pregnancy (14–16). Therefore, a pregnant woman's EER was 2,000 calories plus 400 calories.

The 2006 Food Guide for the Brazilian Population indicates the appropriate portion of food groups that should be consumed daily by a healthy individual, thus served as a basis for the healthy meal plan (17). Calories and home measures were determined using the Home Measurement Table (18).

### Analysis of Iodine in Commercial Salt

To verify iodine concentration in table salt, 13 brands sold in commercial establishments in the city of Viçosa, Minas Gerais, were collected.

The samples were analyzed in the Chemistry Research and Food Analysis Laboratory located in the Food Technology Department of the Federal University of Viçosa (UFV), using the techniques recommended by the Ministry of Health and the Adolfo Lutz Institute manual (19).

For the classification of iodine concentration, iodine levels between 15 and 45 mg/kg were considered adequate as per the criteria of the National Health Surveillance Agency (8).

### Analysis of Iodine in Water

Water samples from 14 Basic Health Units (municipal headquarters) were collected at summer and autumn. Approximately 400 mL of water from each Basic Health Unit was collected in a sterile, hermetically sealed, pre-identified bottle. Iodine concentration was determined by the “Leuco Crystal Violet” Spectrophotometry method in the Chemical Research and Food Analysis Laboratory of the Food Technology Department (UFV) (20–22).

### Statistical Analysis

Descriptive statistics and exploratory data analysis were employed. The results were presented in absolute and relative frequencies. Iodine concentration in salt and drinking water was presented as mean and standard deviation. For dietary iodine intake, the data were presented as median, minimum and maximum values. All the analysis was performed in the Statistical Package for Social Sciences (SPSS) statistical program, version 23.0.

## Results

### Assessment of Dietary Iodine Intake

Considering a feasible healthy eating plan for Brazilian pregnant women, daily iodine intake would be ~71.6 μg (Table 1). Note that cakes and breads were maintained in the food plans despite their undefined iodine content (Dietary Iodine Table) because these foods are frequently eaten by Brazilians.

**Table 1:** Food plan for a pregnant woman.

**Schedule**	**Food**	**Homemade measure**	**Calorie (kcal)**	**Iodine (μg)**
07:00 a.m.	Whole wheat bread	2 Slices (50 g)	140.5	ND
	Mozzarella	1 Slice (20 g)	65.0	0.9
	Coffee with sugar	½ Cup (120 mL)	66.0	2.0
	Papaya	½ Unit (135 g)	48.6	0.5
10:00 a.m	Fruit yogurt	½ Cup (80 ml)	38.5	5.6
	Simple Cake	1 Medium piece (60 g)	263.4	ND
12:00 p.m.	White rice	3 Serving spoons (135 g)	221.4	2.7
	Black beans	1 Medium ladle (140 g)	96.6	2.6
	Chicken filet	1 Medium unit (100 g)	145.0	3.0
	Boiled beet	2 Full tablespoons (40 g)	17.6	0.2
	White cabbage	3 Full tablespoons (30 g)	9.9	0.6
	Tomato	1/2 Medium unit (50 g)	12.0	1.0
	Orange juice	1 Glass cup (240 mL)	182.4	2.4
15:00 p.m.	Banana	2 Units (80 g)	79.2	2.0
	Oat flakes	2 Shallow tablespoons (14 g)	51.9	0.2
	Honey	1 Teaspoon (2 g)	6.2	0.01
17:30 p.m.	Whole milk	1 Glass cup (240 mL)	145.4	35.5
	Coffee with sugar	½ glass cup (120 mL)	66.0	2.0
	French bread	1 Unit (50 g)	134.5	ND
	Mozzarella	1 Slice (20 g)	65.0	0.9
20:00 p.m.	White rice	2 Serving spoons (90 g)	147.6	1.8
	Black beans	1 Medium ladle (140 g)	96.6	2.6
	Boiled Muscle	2 Full tablespoons (60 g)	111.6	0.6
	Boiled English potatoes	2 Full tablespoons (60 g)	51.0	0.7
	Sautéed cabbage	2 Full tablespoons (40 g)	58.4	0.4
	Tomato	1/2 Medium unit (50 g)	12.0	1.0
	Pineapple Juice	1 Glass cup (240 mL)	103.2	2.4
Total			2,435.1 kcal	71.6 μg

*Viçosa, Minas Gerais, 2019*.

*ND, Not Determined—Amount of Iodine Not Determined*.

In the proposed healthy eating plan for schoolchildren, the daily intake of iodine would be ~71.0 μg (Table 2).

**Table 2:** Food plan for a schoolchild.

**Schedule**	**Food**	**Homemade measure**	**Calorie (kcal)**	**Iodine (μg)**
06:30 a.m.	French bread	1 Unit (50 g)	134.5	ND
	Mozzarella	1 Slice (25 g)	65.0	0.9
	Whole milk	½ Glass cup (120 mL)	61.0	14.8
	Coffee with sugar	1 Cup of coffee (50 mL)	33.0	1.0
09:00 a.m.	Banana	2 Units (80 g)	79.2	2.0
11:30 a.m.	White rice	3 Full tablespoons (75 g)	123.0	2.4
	Black beans	1 Small ladle full (65 g)	44.9	1.2
	Roasted pork loin	Small piece (75 g)	216.0	1.6
	Boiled broccoli	2 Tablespoons (20 g)	7.2	0.1
	Tomato	2 Medium slices (15 g)	3.6	0.4
	Lettuce	2 Medium leaves (20 g)	3.8	0.2
	Orange juice	1 Glass cup (165 mL)	125.4	1.6
14:00 p.m.	Melon	1 Medium slice (90 g)	25.2	0.3
16:00 p.m.	Fruit yogurt	½ Glass cup (120 mL)	77.0	11.1
	Simple cake	1 Small slice (30 g)	131.7	ND
18:30 p.m.	White rice	3 Full tablespoons (75 g)	123.0	2.4
	Black beans	1 Small ladle full (65 g)	44.9	1.2
	Boiled chicken egg	1 Medium unit (45 g)	71.1	14.2
	Boiled pineapple	1 Tablespoon (36 g)	25.2	0.05
	Cucumber	4 Small slices (12 g)	2.0	0.1
	Tomato	2 Medium slices (15 g)	3.6	0.4
21:30 p.m.	Whole milk	½ Glass cup (120 mL)	61.0	14.8
	Strawberry	3 Units (36 g)	14.4	0.3
Total			1,475.7 kcal	71.0 μg

*Viçosa, Minas Gerais, 2019*.

*ND, Not Determined—Amount of Iodine Not Determined*.

### Analysis of Iodine Intake From Commercial Salt

Thirteen brands of commercial salt were evaluated. According to the criteria of the National Health Surveillance Agency (8), 69.2% (*n* = 9) of the samples presented iodine concentration within the range of 15–45 mg/kg, 15.4% (*n* = 2) were above standard, and 15.4% (*n* = 2) below standard (Table 3).

**Table 3:** Analysis of iodine concentration in different brands of commercial salt available in local establishments.

**Salt Brands**	**Mean iodine concentration (mg) ±SD**	**Classification**
1	29.41 ± 0.06	Compliant
2	65.20 ± 4.61	Non-compliant
3	38.24 ± 3.38	Compliant
4	38.11 ± 4.59	Compliant
5	15.00 ± 0.00	Compliant
6	25.06 ± 1.39	Compliant
7	20.95 ± 1.06	Compliant
8	58.08 ± 0.43	Non-compliant
9	0.00 ± 0.00	Non-compliant
10	37.11 ± 3.25	Compliant
11	23.00 ± 0.77	Compliant
12	38.25 ± 4.09	Compliant
13	0.00 ± 0.00	Non-compliant

*Viçosa, Minas Gerais, 2019*.

^**^National Health Surveillance Agency legislation = 15–45 mg/iodine per kilogram of salt (8). Mean Iodine Concentration in 1 kilogram of salt. SD, Standard Deviation.

The analysis of iodine content in salt is crucial for estimating iodine intake. With the mean iodine content being 29.88 mg, a salt intake of 5 g per day (Ministry of Health recommendation) results in an iodine ingestion of ~149.4 μg/day (17). However, if we consider 12 g of salt due to the high consumption of processed foods among the Brazilian population (data from Family Budget Survey), iodine ingestion is ~358.5 μg/day (7).

### Analysis of Iodine Concentration in Drinking Water

From the analysis of the water samples collected in the summer, one liter of water contained ~25 μg of iodine. For the water samples collected in autumn, 1 liter of water contained ~14 μg of iodine. Therefore, the drinking water of the Basic Health Units of Viçosa has a mean iodine concentration of ~19.5 μg/liter.

The Institute of Medicine (IOM) recommends that schoolchildren aged 4–8 years should drink ~1.7 liters of water/day, which contributes 33.1 μg to total iodine intake. For pregnant women, the recommended volume is 3 liters, contributing 58.5 μg to total iodine intake (23).

The IOM recommendation is high and often not properly followed by the population. In this case, a realistic water intake would be in milliliters (mL) based on body weight, being 35 mL, and 50–60 mL per kilogram (kg) of body weight, respectively, for healthy adults and children. Thus, the daily intake of iodine would be estimated according to the body weight of each individual (24).

### Daily Iodine Intake

Considering a healthy dietary pattern for pregnant women and schoolchildren, the daily iodine intake of both groups exceeds the recommended levels (Table 4).

**Table 4:** Daily iodine intake of pregnant women and schoolchildren considering a healthy eating pattern.

**Healthy food pattern**	**Pregnant**	**Schoolchildren**
Food plan	71.6 μg	71 μg
Drinking water	58.5 μg	33.1 μg
Consumption of salt	149.4 μg	149.4 μg
Total	279.5 μg	253.5 μg

*Viçosa. Minas Gerais. 2019*.

^**^Healthy intake = 3 liters of water for pregnant women and 1.7 liters for schoolchildren; 5 grams of salt.

## Discussion

According to our theoretical model, the daily iodine intake of pregnant women and schoolchildren is higher than recommended (2). Brazil is currently considered a country which presents more than sufficient iodine intake (23). This classification was based on studies with schoolchildren, and does not reflect the real iodine status of pregnant women.

Based on our model and a healthy eating pattern, schoolchildren would have an iodine intake of 253.5 μg, which is higher than the 120 μg recommended by WHO (2). Corroborating our results, a cross-sectional study carried out in Vespasiano (Minas Gerais state) with 428 schoolchildren showed that 46.7% had more than adequate iodine levels and 20.1% had excess iodine (12). In Ribeirão Preto (São Paulo state), a cross-sectional study evaluated iodine intake among 8-and-10 year old children via urinary iodine, iodine concentration in table salt, and thyroid volume determined by ultrasonography. From the study, 59.5% of the children had iodine levels above 300 μg/L, classified as excessive (24).

For pregnant women following a healthy eating pattern, the calculated recommended intake is also exceeded. However, 279.5 μg is very close to the recommendation of 250 μg. In fact, cooking and food storage can result in possible iodine losses which coupled with a healthy eating pattern can meet the iodine requirement (25).

Pregnant women rarely present excessive iodine status. In a case-control study conducted in Ribeirão Preto (São Paulo state), 9.9% of pregnant women had urinary iodine >250 μg/L, above the requirement (23). In another study conducted in São Paulo, 4.4% of pregnant women had urinary iodine above 250 μg/L (25). A study in Rio de Janeiro reported excess iodine (≥500 μg/L) in 4.5% of pregnant women in Rio de Janeiro (3). As expected, the prevalence of excessive iodine is low due to the high nutritional needs of pregnant women.

Drinking water was not a protective factor against iodine deficiency, since very low iodine values were detected. The level of iodine in water reflects its concentration in the rocks and soils of the region, and varies according to geographic location, where mountainous regions and those far from the sea tend to have lower concentrations of iodine in the soil, water, and food (14).

The mean iodine concentration in the salt samples was within the recommended range and 69.2% of the samples were in accordance with the current legislation (8). However, 30.8% of the salt brands presented iodine concentration below or above the recommended value, which reinforces the importance of effective inspection to ensure compliance with the legislation.

Furthermore, if a schoolchild ingests 5 g of salt per day, he will be consuming 149.4 μg/day of iodine, which already exceeds the recommended daily intake. However, if we consider the current salt intake observed by the Family Budget Survey, of 12 g, even with a healthy pattern, the daily intake of iodine for pregnant women and schoolchildren can reach 488.1 and 462.1 μg, respectively. This worsens the scenario of excessive intake because iodine intake is almost three times (2.98) higher than the recommendation for schoolchildren and almost two times (1.43) higher than that of pregnant women.

If pregnant women consume only 5 g of salt, they will not meet the recommended daily intake of iodine, thus increasing the risk of deficiency. In a cross-sectional study carried out in São Paulo, 57% of pregnant women were diagnosed with iodine deficiency (23). A cross-sectional study carried out in Rio de Janeiro showed that 48.7% of pregnant women were iodine deficient (3). These results demonstrate the importance of further studies to develop effective public policies to control and prevent iodine deficiency.

The strength of this study is the theoretical model of daily iodine intake in pregnant women and schoolchildren, which can guide actions and public policies that targets all forms of iodine consumption. Furthermore, new studies evaluating the nutritional status of iodine will be able to formulate hypotheses about what may be causing changes in the pattern of iodine intake, which, depending on age, alter required daily intake. A limitation of the study is that iodine nutritional status was based on dietary estimates and not urinary iodine concentration, which is the gold standard for assessing a population's nutritional iodine status. Moreover, to assess the concentration of iodine in foods, a table with compiled information from the literature was used. Although the table has not yet been validated in Brazil, it is currently the only table in the country containing iodine content of foods.

## Final Thoughts

The daily iodine intake of schoolchildren and pregnant women was estimated to be above the recommended level, considering a feasible healthy dietary pattern.

With the reduction of the iodization range of salt, the main source of iodine, if schoolchildren consume 5 g of salt per day based on a mean iodine range (30 ppm), they exceed the recommendation. For pregnant women, this does not occur. Therefore, the theoretical model of iodine intake shows the need for current policies to be reviewed with emphasis on the peculiarity of each population group. Moreover, different iodine concentrations were observed in salt brands, which reinforce the importance of a more effective inspection to ensure compliance with the current legislation.

## Data Availability Statement

The original contributions presented in the study are included in the article/supplementary material, further inquiries can be directed to the corresponding author/s.

## Author Contributions

AC contributed significantly to the conception of the paper, its design, data collection, data interpretation, and analysis. SR contributed significantly to the interpretation of data and analysis, participated in the writing and critically revised the paper in a manner sufficient to establish ownership of the intellectual content. MM, EF, ED, ML, SP, MP, RM, and SF critically revised the article in a manner sufficient to establish ownership of the intellectual content. All authors contributed to the article and approved the submitted version.

## Funding

We would like to thank the Coordination of Improvement of Higher Education Personnel - Brazil (CAPES) - Financing Code 001. National Council for Scientific and Technological Development (CNPq), case 408295/2017-1. Foundation of Support and Research of the State of Minas Gerais (FAPEMIG) case APQ-03336-18.

## Conflict of Interest

The authors declare that the research was conducted in the absence of any commercial or financial relationships that could be construed as a potential conflict of interest.

## Publisher's Note

All claims expressed in this article are solely those of the authors and do not necessarily represent those of their affiliated organizations, or those of the publisher, the editors and the reviewers. Any product that may be evaluated in this article, or claim that may be made by its manufacturer, is not guaranteed or endorsed by the publisher.
